# Downregulation of YAP Activity Restricts P53 Hyperactivation to Promote Cell Survival in Confinement

**DOI:** 10.1002/advs.202302228

**Published:** 2023-06-02

**Authors:** Farnaz Hemmati, Ayuba Akinpelu, Jiyeon Song, Farshad Amiri, Anya McDaniel, Collins McMurray, Alexandros Afthinos, Stelios T. Andreadis, Andrew V. Aitken, Vinicia C. Biancardi, Sharon Gerecht, Panagiotis Mistriotis

**Affiliations:** ^1^ Department of Chemical Engineering Auburn University Auburn AL 36849 USA; ^2^ Department of Biomedical Engineering Duke University Durham NC 27708 USA; ^3^ Cooper Medical School of Rowan University Camden NJ 08103 USA; ^4^ Departments of Chemical and Biological Engineering The State University of New York Buffalo NY 14260 USA; ^5^ Department of Biomedical Engineering University at Buffalo The State University of New York Buffalo NY 14228 USA; ^6^ Center of Excellence in Bioinformatics and Life Sciences Buffalo NY 14203 USA; ^7^ Center for Cell Gene and Tissue Engineering (CGTE) University at Buffalo The State University of New York Buffalo NY 14260 USA; ^8^ Department of Anatomy Physiology and Pharmacology College of Veterinary Medicine Auburn University Auburn AL 36849 USA; ^9^ Center for Neurosciences Initiative Auburn University Auburn AL 36849 USA

**Keywords:** 3D confinement, apoptosis, cell mechanosensing, cell migration, P53, YAP

## Abstract

Cell migration through confining three dimensional (3D) topographies can lead to loss of nuclear envelope integrity, DNA damage, and genomic instability. Despite these detrimental phenomena, cells transiently exposed to confinement do not usually die. Whether this is also true for cells subjected to long‐term confinement remains unclear at present. To investigate this, photopatterning and microfluidics are employed to fabricate a high‐throughput device that circumvents limitations of previous cell confinement models and enables prolonged culture of single cells in microchannels with physiologically relevant length scales. The results of this study show that continuous exposure to tight confinement can trigger frequent nuclear envelope rupture events, which in turn promote P53 activation and cell apoptosis. Migrating cells eventually adapt to confinement and evade cell death by downregulating YAP activity. Reduced YAP activity, which is the consequence of confinement‐induced YAP1/2 translocation to the cytoplasm, suppresses the incidence of nuclear envelope rupture and abolishes P53‐mediated cell death. Cumulatively, this work establishes advanced, high‐throughput biomimetic models for better understanding cell behavior in health and disease, and underscores the critical role of topographical cues and mechanotransduction pathways in the regulation of cell life and death.

## Graphical abstract

This study employs novel microfluidic devices to demonstrate how migrating cells respond and adapt to long‐term confinement. Constant exposure to confined microenvironments that deform the nucleus can reduce cell viability. However, YAP translocation to the cytoplasm helps cells adapt to confinement by reducing the frequency of nuclear envelope rupture events and preventing P53‐mediated cell death.

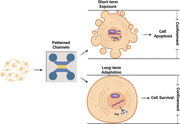

## 1 Introduction

Cell migration is a fundamental cellular phenomenon that plays a pivotal role in pathophysiological events such as cancer metastasis. In vivo, migrating cells must move through confining three dimensional (3D) topographies, including microvessels with a diameter smaller than the size of cells,^[^ ^1^ ^]^ narrow (2–5 µm‐sized) gaps between endothelial cells,^[^ ^2^ ^]^ micropores, which have a minimum diameter of ≈1 µm,^[^ ^3^ ^]^ and fiber‐ and channel‐like tracks, which have a minimum width of ≈3 µm.^[^ ^4^ ^]^ It is established that confining microenvironments can physically deform the cell and its nucleus, ultimately inducing nuclear envelope rupture (NER) events, DNA damage, and genomic instability.^[^ ^5^ ^]^ Despite these detrimental phenomena, transient exposure to confinement, as a result of migration through narrow constrictions, rarely triggers cell death because cells have sufficient mechanisms in place to repair such nuclear damages.^[^ ^5a,b^ ^]^ In contrast, entrapment of migrating cells in confining microvessels is associated with reduced cell viability.^[^ ^1^, ^6^ ^]^

To date, the impact of long‐term confinement on cell survival remains unclear. To address this gap in knowledge, it is necessary to culture cells for prolonged periods in physiologically relevant and well‐defined microenvironments that enable high‐throughput investigation of confinement‐dependent responses. However, published cell confinement assays, such as micropatterned lines, 2D micropatterned substrates, or uni‐axial compression, constrain cells only in one or two dimensions,^[^ ^7^ ^]^ in contrast to what is typically observed in vivo. Microniches, which permit cell entrapment in microenvironments of prescribed stiffness, have dimensions that are larger than the average cell diameter and thereby cannot induce tight confinement.^[^ ^8^ ^]^ Cells can experience complete 3D confinement upon encapsulation in natural or synthetic hydrogels; yet, these assays suffer from numerous limitations, including a narrow range of pore sizes, which are often smaller than those encountered by migrating cells in vivo.^[^ ^3^, ^7^, ^9^ ^]^ Furthermore, it is challenging to systematically tune the hydrogel porosity independent of other physical parameters (e.g., substrate stiffness).^[^ ^7^ ^]^ Although recent advances in photopatterning enable the generation of channels within a 3D matrix with relatively high resolution in the *x‐*‐*y* and *z* plane, this method is low‐throughput.^[^ ^10^ ^]^

Here, we have employed microfluidics and light‐induced patterning to develop an innovative high‐throughput cell confinement device that circumvents the limitations of previous assays. This technology enables fine‐tuning of the degree and geometry of confinement, permits prolonged culture of single cells in tightly confining microenvironments coated with extracellular matrix (ECM) molecules, and allows real‐time monitoring of cell behavior and function at maximum optical resolution. Using this assay with sophisticated live‐cell reporters, we show that YAP‐dependent signaling regulates P53 activity, thereby controlling cell life and death in confinement. Overall, these results underscore the importance of topographical cues and mechanotransduction pathways in the regulation of cell survival.

## 2 Results

### 2.1 Photopatterning and Microfluidics Enable Cell Entrapment in Microchannels of Prescribed Dimensions

**Figure 1:**
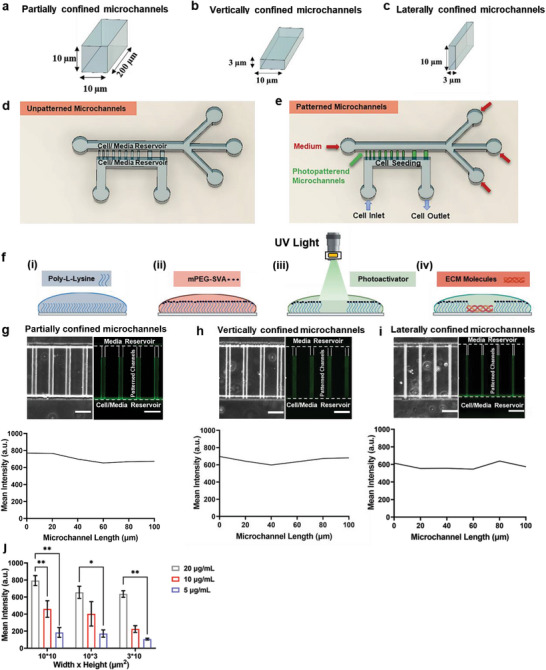
ECM deposition on microchannel walls using photopatterning. a–c) 3D representation of a) partially (*W* × *H*: 10 × 10 µm^2^), b) vertically (*W* × *H*: 10 × 3 µm^2^), and c) laterally (*W* × *H*: 3 × 10 µm^2^) confined microchannels. d,e) Schematic representation of our microfluidic device d) without or e) with photopatterned microchannels. f) Steps of photopatterning (modified from^[^ ^40^ ^]^): i) coating with PLL, ii) surface passivation using mPEG‐SVA, iii) removal of PLL‐mPEG‐SVA using UV light and the photoactivatable reagent PLPP, iv) coating of the surface with ECM molecules. g–i) Representative images (top) of g) partially, h) vertically, and i) laterally confined microchannels photopatterned with Collagen I‐FITC and mean intensity values of the deposited Collagen I‐FITC on those microchannels (bottom). Scale bar, 50 µm. j) Mean intensity of Collagen I‐FITC photopatterned on the walls of partially, vertically, and laterally confined microchannels. Collagen I‐FITC concentration: 5–20 µg mL^−1^ (*n* ≥ 40 channels from at least four independent experiments). Values are mean ± S.E.M. *p < 0.05, ** *p* ≤ 0.01.

We integrated microfluidics with light‐induced patterning to fabricate devices that allowed us to study how long‐term 3D confinement affected cell behavior. Using standard multilayer photolithography and replica molding, we first built polydimethylsiloxane (PDMS)‐based microfluidic devices^[^ ^11^ ^]^ that consisted of an array of parallel microchannels with dimensions that mimic the size of channel‐like tracks or microvessels encountered by migrating cells in vivo.^[^ ^1^, ^4^ ^]^ Specifically, we fabricated partially (Width(*W*) × Height(*H*) = ≈10 × 10 µm^2^), vertically (*W* × *H* = ≈10 × 3 µm^2^), and laterally (*W* × *H* = ≈3 × 10 µm^2^) confined microchannels (**Figure** **1**a–c), the dimensions of which were verified using a laser profilometer (Figure S1a, Supporting Information). Vertically and laterally confined microchannels were chosen because they had the same cross‐sectional area but different aspect ratios, enabling us to investigate how different microchannel geometries influence cell behavior.^[^ ^11a^ ^]^ All microchannels had a constant length (200 µm). Perpendicular to these microchannels were two larger, 2D‐like channels that served as a cell and media reservoir (Figure 1d). Next, our devices were treated with poly‐l‐lysine (PLL) followed by methoxy poly(ethylene glycol) (mPEG)–succinimidyl valerate (mPEG‐SVA) deposition to create an antifouling layer that prevents cells and ECM molecules from adhering to the device surface.^[^ ^11a^ ^]^ By applying the PRIMO photopatterning technology and the photoactivatable reagent PLPP,^[^ ^12^ ^]^ we degraded the anti‐adhesive layer that lined the microchannel walls in order to coat them with ECM proteins (Figure 1e,f(i–iv)). Because photopatterning the entire length of the microchannel might trigger cell migration toward the uppermost 2D‐like channel and thus suppress cell entrapment, only a portion (≈75%) of each microchannel was coated with ECM. To visualize ECM protein deposition, Collagen type I‐conjugated with fluorescein isothiocyanate (FITC) was photopatterned on the walls of partially, vertically, and laterally confined microchannels. Widefield and confocal fluorescence microscopy demonstrated uniform Collagen I‐FITC (20 µg mL^−1^) coating throughout the microchannel walls irrespective of microchannel size or geometry (Figure 1g–i and Figure S1b, Supporting Information). Moreover, the amount of collagen deposition decreased with reducing Collagen I‐FITC concentration, and these results were independent of microchannel dimensions (Figure 1j). Of note, at a given Collagen I‐FITC concentration, the deposited collagen was similar between different geometries (Figure S1c, Supporting Information). At the same time, unpatterned regions of the device (e.g., cell and media reservoir or unpatterned microchannels) only showed background fluorescence (Figure S1d, Supporting Information).

**Figure 2:**
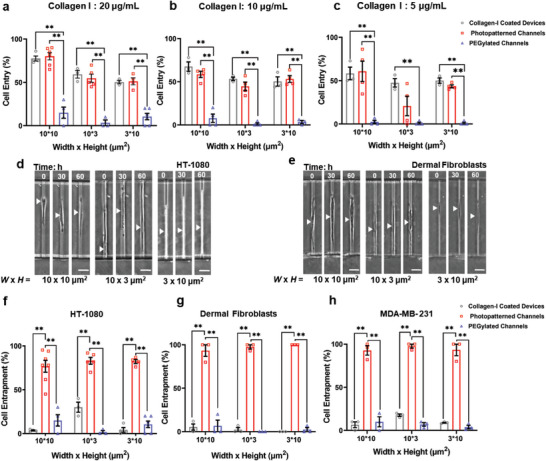
Successful entrapment of cancerous and non‐cancerous cells in partially, vertically, and laterally confined microchannels. a–c) Percentage of cell entry into partially, vertically, and laterally confined microchannels coated with different concentrations of Collagen I (5–20 µg mL^−1^). Cell entry was evaluated 2 h after cell seeding and in the presence of chemoattractant molecules. Collagen I‐coated devices and PEGylated devices served as positive and negative controls respectively (*n* ≥ 45 cells from at least three independent experiments). Values are mean ± S.E.M. ** *p* ≤ 0.01. d,e) Image sequence of d) human HT‐1080 fibrosarcoma cells or e) human dermal fibroblasts entrapped in partially, vertically, and laterally confined microchannels. The arrowhead indicates cell position. Scale bar: 20 µm. f–h) Entrapment efficiency of f) HT‐1080 cells, g) dermal fibroblasts, and h) MDA‐MB‐231 breast cancer cells in partially, vertically, and laterally confined microchannels coated with 20 µg mL^−1^ Collagen I after 18 h. Collagen I‐coated devices and PEGylated devices served as controls (*n* ≥ 45 cells from at least three independent experiments). Values are mean ± S.E.M. **p ≤ 0.01.

To introduce cells into the devices, we added a suspension of HT‐1080 fibrosarcoma cells to the inlet wells. We thus generated a pressure differential between the inlet and outlet wells, which drove cell flow through the lowermost 2D‐like channel (Figure 1e). Although cells failed to adhere to channels treated with PLL‐mPEG‐SVA, they attached adjacent to the entrances of Collagen I photopatterned microchannels (Figure S2a, Supporting Information). During the first 2 h after cell seeding, a fraction of cells (20–40%) ingressed successfully into these microchannels (Figure S2b, Supporting Information). Of note, coating the whole device with Collagen I (hereafter termed Collagen I‐coated devices) produced similar percentages of cell entry (Figure S2b, Supporting Information). To improve cell entry efficiency, we generated a chemical gradient by introducing chemoattractant molecules (10% v/v fetal bovine serum [FBS]) into the uppermost 2D‐like channel and serum‐free medium into the lower one (Figure 1e). In the presence of chemotaxis, cell entry into microchannels photopatterned with 20 µg mL^−1^ Collagen I reached ≈55–75% after 2 h (**Figure** **2**a) and ≈70–90% after 18 h (Figure S2c, Supporting Information). Photopatterning of vertically or laterally confined microchannels with lower concentrations of Collagen I (10 or 5 µg mL^−1^) reduced cell entry and led to more variability between experiments (Figure 2b,c). As a result, all remaining experiments were conducted using 20 µg mL^−1^ Collagen I.

Next, we quantified the fraction of cells that remained inside microchannels following cell entry. Our analysis was focused only on microchannels that contained individual cells. Using both cancerous (human HT‐1080 fibrosarcoma cells, human MDA‐MB‐231 breast cancer cells) and non‐cancerous cells (human dermal fibroblasts ) as cell models, we found that the efficiency of cell entrapment in photopatterned microchannels reached ≈80–95% and was independent of their geometry (Figure 2d–h and Videos S1–S3). In contrast, Collagen I‐coated devices, which facilitated cell migration through microchannels (Figure S2d, Supporting Information), could not entrap cells in confined environments (Figure 2f–h). Moreover, PEGylated devices (i.e., devices treated only with PLL‐mPEG‐SVA) hindered the ability of cells to attach and spread and thus significantly suppressed cell entry (Figure 2a–c and Figure S2b,c, Supporting Information) and entrapment (Figure 2f–h). In sum, integrating microfluidics with photopatterning enables the development of a platform technology that permits prolonged culture of single cells in microchannels of prescribed dimensions.

### 2.2 Cell Division and Death in Confinement

**Figure 3:**
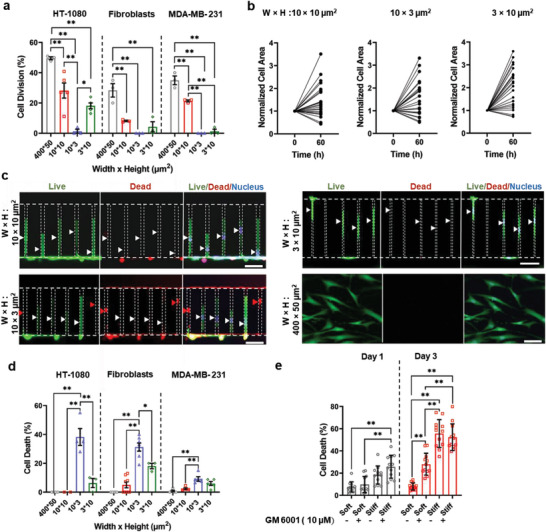
3D confinement regulates cell division and cell death. a) Percentage of HT‐1080 cells, fibroblasts, and MDA‐MB‐231 breast cancer cells that divided inside partially, vertically, and laterally confined microchannels over the course of 18 h (*n* ≥ 45 cells from at least three independent experiments). 400 × 50 µm^2^ channels served as a 2D control. Data are mean ± S.E.M. ** *p* ≤ 0.01. b) Longitudinal area of dermal fibroblasts (normalized to *t* = 0 h) as a function of time inside partially, vertically, and laterally confined microchannels (*n* ≥ 25 cells from at least three independent experiments). c) Live–dead staining of dermal fibroblasts in partially, vertically, and laterally confined microchannels after 3 days of entrapment. The white and red arrowheads show live and dead cells, respectively. Scale bar: 50 µm. d) Percentage of HT‐1080 cells, dermal fibroblasts, and MDA‐MB‐231 breast cancer cells that died inside partially, vertically, and laterally confined microchannels after 3 days of entrapment (*n* ≥ 45 cells from at least three independent experiments). 400 × 50 µm^2^ channels served as a 2D control. Data are mean ± S.E.M. **p ≤ 0.01, *p < 0.05. e) Percentage of cell death inside soft or stiff methacrylated collagen hydrogels on days 1 and 3. HT‐1080 cells were treated with an MMP inhibitor (GM 6001; 10 µm) or vehicle control (VC; *n* ≥ 2000 cells from three independent experiments). Data are mean ± SD; ** *p* ≤ 0.01.

Next, we quantified cell division and viability inside partially, vertically, and laterally confined microchannels. As a 2D control, we employed Collagen I‐coated channels with a cross‐sectional area of 400 × 50 µm^2^. In line with prior reports,^[^ ^13^ ^]^ the percentage of cells that divided over the course of 18 h reduced in confining microenvironments relative to the 2D control. This observation held true for HT‐1080 fibrosarcoma cells, dermal fibroblasts, and MDA‐MB‐231 breast cancer cells (**Figure** **3**a). In addition, we detected reduced cell proliferation in confinement in later phases of cell entrapment (e.g., day 2 and 3; Figure S3a, Supporting Information). Concurrently, the majority (>50%) of non‐dividing cells displayed an increase in their longitudinal area after 60 h in confinement (Figure 3b). Although cells maintained high viability in unconfined and partially confined microenvironments, vertical, but not lateral, confinement induced cell death, especially in HT‐1080 cells and dermal fibroblasts (Figure 3c,d). These data ruled out the possibility that cell death in vertical confinement was due to nutrient deprivation because laterally and vertically confined microchannels had the same cross‐sectional area (30 µm^2^). Additionally, by monitoring the diffusion of bovine serum albumin (BSA) conjugated with FITC, we verified that vertically confined cells had access to nutrients (Figure S3b, Supporting Information). It is noteworthy that MDA‐MB‐231 breast cancer cell viability remained high (≥≈90%) in confinement (Figure 3d), irrespective of microchannel geometry. These data suggest that susceptibility to confinement‐induced cell death varies between cell lines.

To extend our findings from stiff PDMS‐based microchannels to more deformable biological environments, we used 3D collagen‐based hydrogel matrices with varying stiffness and pore sizes. Encapsulation of HT‐1080 fibrosarcoma cells in soft, ≈240 Pa hydrogels with a mean pore size of ≈1.5 µm^2^ (Figure S3c–e, Supporting Information) supported cell proliferation, spreading, and survival during 3 days of culture (Figure 3e and Figure S3f,g, Supporting Information). In contrast, stiff hydrogels (≈2 kPa) with reduced pore size (Figure S3c–e, Supporting Information) compromised cell elongation (Figure S3g, Supporting Information), hindered cell growth (Figure S3f,g, Supporting Information), and promoted cell death (Figure 3e). Importantly, treatment of cells in soft matrices with GM 6001 (10 µm), a matrix metalloproteinase (MMP) inhibitor that prevented cells from widening pores,^[^ ^5a^ ^]^ resulted in similar outcomes to those observed in stiff hydrogels with smaller pores; reduced number of cells (Figure S3f,g, Supporting Information) and increased cell death (Figure 3e) on day 3 of encapsulation. These findings suggest that cell death can occur in confined microenvironments with physiologically relevant mechanics.

### 2.3 P53 Activation in Vertical Confinement Triggers Cell Apoptosis

**Figure 4:**
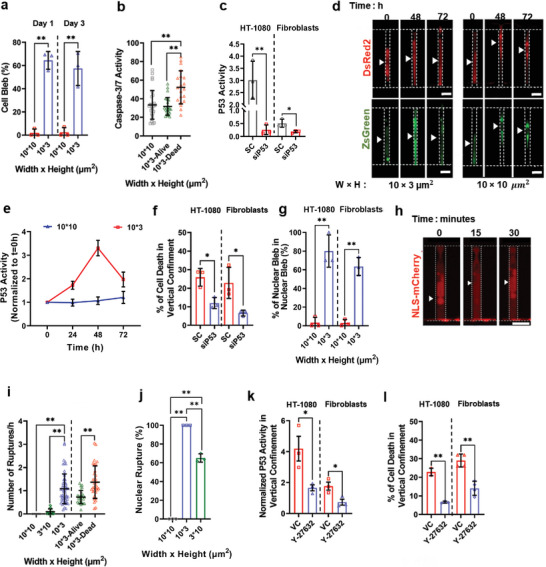
Confinement‐induced stress on the nucleus promotes P53‐dependent gene expression and apoptosis. a) Percentage of dermal fibroblasts exhibiting membrane blebbing in partially versus vertically confined microchannels after 1 or 3 days of entrapment (*n* ≥ 45 from three independent experiments). b) Caspase‐3/7 activity in partially confined fibroblasts or in vertically confined fibroblasts that remained alive or died (*n* ≥ 20 cells from three independent experiments). Caspase 3/7 activity was assessed using the NucView dye. Data are mean ± SD; **p ≤ 0.01. c) Quantification of P53 activity in HT‐1080 cells and dermal fibroblasts. Cells were transfected with a scramble (SC) or siP53 sequence (*n* ≥ 200 from three independent experiments). Data are mean ± S.E.M. **p <0.01, *p < 0.05. d) Image sequence showing P53 activity in partially and vertically confined dermal fibroblasts. Scale bar: 20 µm. e) P53 activity (normalized to *t* = 0 h) as a function of time in partially and vertically confined dermal fibroblasts (*n* ≥ 40 cells from four independent experiments). Data are mean ± S.E.M. *p < 0.05. f) Percentage of cell death in vertically confined HT‐1080 cells or dermal fibroblasts transfected with SC or siP53 sequence. Cell death was assessed 3 days after cell entry into microchannels using live–dead staining (*n* ≥ 45 cells from three independent experiments). Data are mean ± S.E.M. *p < 0.05. g) Percentage of dermal fibroblasts or LifeAct‐GFP‐/H2B‐mCherry‐labeled HT‐1080 cells exhibiting nuclear blebbing in partially versus vertically confined microchannels after 1 day of entrapment (*n* ≥ 45 from three independent experiments). Data are mean ± S.E.M. **p < 0.01. h) Image sequence of a representative NLS‐mCherry‐labeled HT‐1080 cell experiencing a transient nuclear rupture in vertically confined microchannels. Arrowhead indicates nuclear position. Scale bar: 20 µm. i) Number of nuclear ruptures per hour in partially, vertically, or laterally confined HT‐1080 cells labeled with NLS‐mCherry. The frequency of nuclear ruptures was also quantified in vertically confined cells that remained alive or died (*n* ≥ 30 cells from three independent experiments). Data are mean ± SD; ** *p* ≤ 0.01. j) Percentage of HT‐1080 cells experiencing NER in partially, vertically, or laterally confined microchannels (*n* ≥ 30 cells from three independent experiments). Data are mean ± S.E.M. **p < 0.01. k) P53 activity (normalized to *t* = 0) in dermal fibroblasts or HT‐1080 cells 48 h after cell entry into vertically confined microchannels. Cells were treated with VC or ROCK inhibitor (10 µm; Y‐27632) (*n* ≥ 30 cells from three independent experiments). Data are mean ± S.E.M. *p < 0.05. l) Percentage of cell death in vertically confined dermal fibroblasts or HT‐1080 cells treated with VC or ROCK inhibitor (10 µm; Y‐27632). Cell death was assessed 3 days after cell entry into microchannels using live–dead staining (*n* ≥ 60 cells from at least three independent experiments). Data are mean ± S.E.M. ** *p* ≤ 0.01.

To identify the mechanism by which confinement promotes cell death, we examined the phenotype of dermal fibroblasts or HT‐1080 cells in partially and vertically confined microchannels. We detected membrane blebbing in ≈60% of vertically confined fibroblasts after 1 or 3 days of entrapment (**Figure** **4**a and Figure S4a, Supporting Information). The extent of membrane blebbing was more pronounced in vertically confined HT‐1080 cells; ≈80% of cells displayed the blebbing phenotype after a day of entrapment (Figure S4b, Supporting Information). Membrane blebs were absent in partially confined microchannels, regardless of the cell type (Figure 4a and Figure S4b, Supporting Information). Next, we monitored the behavior of HT‐1080 cells expressing LifeAct‐GFP (actin) and H2B‐mCherry (histone 2B) over 18 h using fluorescent time‐lapse movies. We found that the percentage of fragmented nuclei increased as a function of time, reaching ≈20% after 18 h (Figure S4c,d, Supporting Information). Because cell blebbing and nuclear fragmentation are indicative of cell apoptosis,^[^ ^14^ ^]^ we stained human fibroblasts with NucView to monitor caspase‐3/7 activation in real‐time. Fluorescence imaging demonstrated that most vertically confined cells increased caspase‐3/7 activity during cell death. In contrast, vertically confined cells that remained alive or partially confined cells failed to activate these proteases (Figure 4b and Figure S4e, Supporting Information). These data reveal that vertical confinement compromises cell viability primarily through the induction of apoptosis.

The vast majority of MDA‐MB‐231 breast cancer cells survived in confinement (Figure 3d). However, in contrast to HT‐1080 cells^[^ ^14^ ^]^ and dermal fibroblasts, MDA‐MB‐231 cells express a mutant P53 gene,^[^ ^15^ ^]^ which may confer resistance to confinement‐induced cell apoptosis.^[^ ^16^ ^]^ To assess the effects of confinement on P53 activity, we employed a lentiviral dual promoter (LVDP) vector that we had previously optimized.^[^ ^17^ ^]^ This vector contains two fluorescent reporters: 1) DsRED2, which is expressed under the constitutively active promoter human phosphoglycerate kinase and is indicative of the number of viral copies per cell, and 2) ZsGreen, which is driven by the P53 binding motif (P53‐Response element (RE)) (Figure S4f, Supporting Information).

By quantifying the green and red fluorescence intensity (GFI and RFI) for each cell at different time points and taking the ratio GFI/RFI, we were able to monitor in real‐time the dynamics of P53 activation in confinement independent of transduction efficiency. To verify that our reporter worked as expected, we showed that treatment with puromycin (1 µg mL^−1^)^[^ ^18^ ^]^ significantly increased P53 transcriptional activity (Figure S4g,h, Supporting Information), whereas knockdown of P53 markedly suppressed P53 transcriptional activity (Figure 4c and Figure S4i,j, Supporting Information). Fluorescence imaging also revealed that P53 activity was elevated in vertically, but not in partially confined microchannels, reaching a maximum on day 2 and decreasing by day 3 (Figure 4d,e). Interestingly, a fraction of vertically confined cells (34%) did not upregulate P53‐RE more than 1.3‐fold (Figure S4k, Supporting Information), presumably due to inherent cell‐to‐cell differences. In line with data obtained using MDA‐MB‐231 breast cancer cells (Figure 3d), human fibroblasts or HT‐1080 cells treated with siP53 showed increased viability in vertical and partial confinement (Figure 4f and Figure S4l, Supporting Information). Similar findings were noted using an inhibitor that blocked P53 transcriptional activity (Pifithrin‐*α*; 20 µm)^[^ ^19^ ^]^ (Figure S4m, Supporting Information).

Confined human fibroblasts and HT‐1080 cells experience NER events, which in turn trigger DNA damage responses.^[^ ^15^ ^]^ Because DNA damage can lead to P53 activation,^[^ ^16^ ^]^ we examined the potential involvement of NER in cell death regulation. Fibroblasts or HT‐1080 cells entrapped in vertically confining microchannels formed pronounced nuclear envelope blebs (Figure 4g and Figure S4a, Supporting Information), which are known to precede or coincide with NER events.^[^ ^5^, ^11^ ^]^ By employing an established fluorescent reporter protein (mCherry fused to a nuclear localization sequence [NLS]) that leaks into the cytoplasm when the nuclear envelope integrity is compromised,^[^ ^5a,b^ ^]^ we detected frequent NER events in ≈100% of vertically confined HT‐1080 cells (Figure 4h–j). The severity of NERs was more pronounced in entrapped cells that ultimately died compared to cells that survived (Figure 4i). In contrast, the frequency of NER events and the fraction of cells experiencing NER were significantly reduced in partially or laterally confined microchannels (Figure 4i,j) that supported cell survival (Figure 3d). Suppression of nuclear blebbing and NER via treatment with the Rho‐associated protein kinase (ROCK) inhibitor Y‐27632^[^ ^11b^ ^]^ (10 µm) markedly reduced confinement‐induced P53 activation and cell death in HT‐180 cells and fibroblasts (Figure 4k,l and Figure S4n, Supporting Information). Conversely, knockdown of the endosomal sorting complexes required for transport III subunit CHMP2A (Figure S4o, Supporting Information) that contributed to nuclear envelope resealing during late anaphase^[^ ^20^ ^]^ and following NER^[^ ^5a,b^ ^]^ increased cell death in both partially and vertically confined microchannels (Figure S4p, Supporting Information). Overall, these findings suggest that vertical confinement promotes the frequent loss of nuclear envelope integrity, which in turn induces P53‐dependent DNA damage responses and, thereby, cell apoptosis.

### 2.4 YAP Exit to the Cytoplasm Promotes Cell Adaptation to Confinement by Downregulating the Incidence of NER and P53 Activity

**Figure 5:**
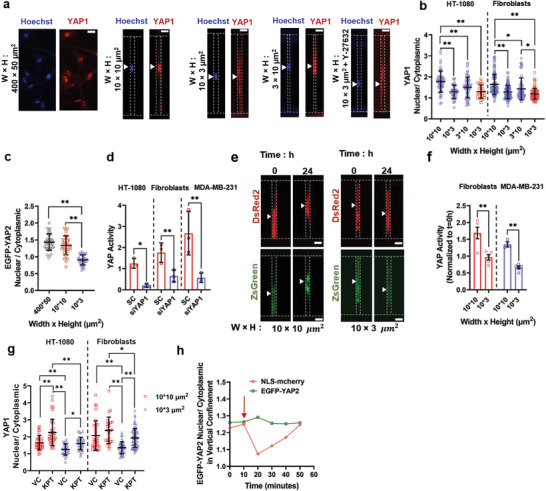
Confinement‐induced YAP exit to the cytoplasm downregulates YAP activity. a) Representative images showing YAP1 localization in dermal fibroblasts cultured on 2D surfaces (400 × 50 µm^2^ channels), as well as in partially, vertically, or laterally confined microchannels. Cells were treated with VC or ROCK inhibitor (10 µm; Y‐27632). Scale bar: 15 µm. b) Nuclear to cytoplasmic ratio of YAP1 in dermal fibroblasts or HT‐1080 cells immediately after cell entry into partially, vertically, or laterally confined microchannels (*n* ≥ 45 cells from at least four independent experiments). Cells were treated with VC (blue circles) or Y‐27632 (red circles). Data are mean ± SD; *p <0.05, **p <0.01. c) Nuclear to cytoplasmic ratio of EGFP‐YAP2 in HT‐1080 cells immediately after cell entry into partially or vertically confined microchannels. 400 × 50 µm^2^ channels served as a 2D control (*n* ≥ 30 from two independent experiments). Data are mean ± SD; **p < 0.01. d) Quantification of YAP transcriptional activity in MDA‐MB‐231 cells, HT‐1080 cells, or dermal fibroblasts transfected with SC or siYAP1 (*n* ≥ 200 from three independent experiments). Data are mean ± S.E.M. *p <0.05, **p < 0.01. e) Image sequence showing YAP activity in partially or vertically confined dermal fibroblasts immediately or ≈24 h after cell entry into microchannels. Scale bar: 10 µm. (f) YAP activity (normalized to *t* = 0 h) in partially and vertically confined dermal fibroblasts or partially and vertically confined MDA‐MB‐231 breast cancer cells ≈24 h after cell entry into microchannels (*n* ≥ 30 from three independent experiments). Data are mean ± S.E.M. **p < 0.01. g) Nuclear to cytoplasmic ratio of YAP1 in dermal fibroblasts or HT‐1080 cells immediately after cell entry into partially or vertically confined microchannels. Cells were treated with VC or the Exportin 1 inhibitor KPT‐330 (10 µm) (*n* ≥ 45 from three independent experiments). Data are mean ± SD; *p <0.05, **p < 0.01. h) Nuclear to cytoplasmic ratio of NLS‐mCherry and EGFP‐YAP2 in a vertically confined, KPT‐330‐treated HT‐1080 cell experiencing a NER event.

A fraction of fibroblasts and HT‐1080 cells (≈60–70%) was able to adapt to vertical confinement and survive (Figure 3c,d). Additionally, the activity of P53 decreased on day 3 (Figure 4e), suggesting the existence of a mechanism that acts to restrict activation of P53‐dependent pro‐apoptotic pathways. Using immunofluorescence, we found that the mechanotransducer YAP1 translocated to the cytoplasm of HT‐1080 cells and fibroblasts immediately after cell entry into vertically, but not into partially, confined microchannels (**Figure** **5**a,b). Increased YAP1 cytoplasmic localization was also observed after 24 h of entrapment (Figure S5a, Supporting Information). The observed change in YAP1 localization was not an artifact of the immunocytochemistry procedure because the nuclear‐to‐cytoplasmic ratio of the nuclear marker Histone H3 was similar between 2D and vertical confinement (Figure S5b, Supporting Information). Furthermore, we transfected HT‐1080 cells with EGFP‐YAP2 to monitor YAP2 localization in real‐time.^[^ ^21^ ^]^ Similar to YAP1 (ref. [22] and Figure 5a,b), EGFP‐YAP2 translocated to the cytoplasm on soft (0.2 kPa) relative to stiff (12 kPa) collagen I‐coated polyacrylamide gels (Figure S5d–f, Supporting Information) and accumulated in the cytoplasm of vertically, but not partially, confined cells (Figure 5c).

YAP1 and YAP2 can regulate gene expression by associating with TEAD transcription factors.^[^ ^23^ ^]^ Thus, we subcloned the YAP/TEAD‐RE (ACATTCCA)^[^ ^22^ ^]^ into the LVDP vector to quantify YAP transcriptional activity. We verified the validity of this reporter by demonstrating that YAP/TEAD‐RE was significantly downregulated in cells plated on soft (0.2 kPa) Collagen I‐coated polyacrylamide gels (Figure S5g,h, Supporting Information)^[^ ^22^ ^]^ and in YAP1 knockdown cells (Figure 5d and Figure S5i–k, Supporting Information). Quantification of YAP transcriptional activity in confinement revealed that ≈24 h after cell entry into microchannels, vertically confined fibroblasts displayed reduced YAP‐dependent gene expression relative to partially confined fibroblasts (Figure 5e,f). Interestingly, a similar reduction was seen in MDA‐MB‐231 cells that carry a mutant P53 gene (Figure 5f), suggesting that P53 did not mediate the observed decrease in YAP activity.

We next investigated how confinement altered the subcellular distribution of YAP. Given that prior work has established the key role of Exportin 1 in YAP nuclear export,^[^ ^24^ ^]^ we treated partially and vertically confined cells with an Exportin 1 inhibitor (KPT‐330; 10 µm). Although treatment with KPT‐330 increased the nuclear levels of YAP1 in both partial and vertical confinement, this intervention failed to abrogate differences between the two confinement types (Figure 5g). Thus, additional mechanisms contributing to YAP nuclear exit must exist. We hypothesized that if nuclear envelope ruptures were involved in triggering YAP translocation to the cytoplasm, then lateral confinement, which suppressed NER events (Figure 4i,j), would promote nuclear accumulation of YAP. Instead, we found that lateral confinement increased the cytoplasmic fraction of YAP1 (Figure 5a,b). We also treated cells with the ROCK inhibitor Y‐27632, which suppressed nuclear blebbing and NER events.^[^ ^11b^ ^]^ This intervention failed to block confinement‐induced YAP1 translocation to the cytoplasm (Figure 5a,b), arguing that NER was not involved in YAP1 nuclear exit. However, Y‐27632 also triggered YAP1 escape in cells cultured on unconfined, 2D surfaces (Figure S5c, Supporting Information) where NER events were rarely detected. These data suggest that the RhoA/ROCK pathway controls YAP nucleocytoplasmic transport independent of NER events, presumably by suppressing Exportin 1‐mediated YAP1 nuclear export.^[^ ^24^ ^]^ Last, we performed live‐cell imaging of HT‐1080 cells co‐expressing EGFP‐YAP2 and NLS‐mCherry to assess whether changes in the subcellular distribution of YAP were directly associated with NER events. We carried out this experiment in vertically confined, KPT‐330‐treated cells because they a) displayed mostly nuclear EGFP‐YAP2 localization (Figure 5g) and b) allowed us to exclude possible confounding effects of active nuclear export on YAP exit to the cytoplasm. Time‐lapse fluorescent microscopy showed that the EGFP‐YAP2 subcellular distribution remained mostly unchanged following a NER event (Figure 5h and Figure Sl–o). Collectively, our findings reveal that YAP exit to the cytoplasm can be regulated by active nuclear export mechanisms but not by NER.

**Figure 6:**
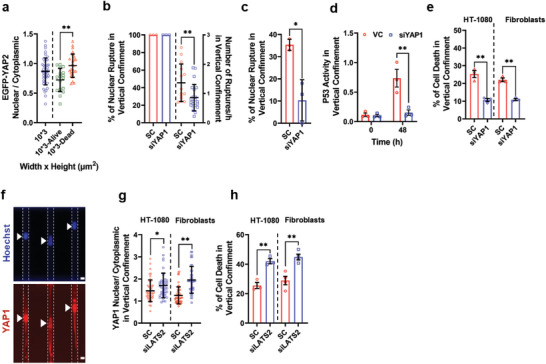
Accumulation of YAP in the cytoplasm supports cell survival in confinement. a) Nuclear to Cytoplasmic ratio of EGFP‐YAP2 in vertically confined HT‐1080 cells that remained alive or died (*n* ≥ 17 from three independent experiments). Data are mean ± SD; **p < 0.01. b) Percentage of vertically confined HT‐1080 cells tagged with NLS‐mCherry experiencing NER as well as the frequency of nuclear ruptures in these cells. Cells were transfected with SC or siYAP1 sequence (*n* ≥ 30 cells from three independent experiments). Data are mean ± S.E.M (% of nuclear rupture) or ± SD; (number of ruptures per hour). **p < 0.01. c) Percentage of dermal fibroblasts experiencing NER in vertically confined microchannels. Cells were transfected with a SC and siYAP1 sequence (*n* ≥ 30 cells from three independent experiments). Data are mean ± S.E.M. *p < 0.01. d) P53 activity in vertically confined dermal fibroblasts transfected with SC or siYAP1 sequence. P53 activity was quantified immediately (*t* = 0 h) or 48 h after cell entry into vertical microchannels (*n* ≥ 40 cells from four independent experiments). Data are mean ± S.E.M. **p < 0.01. e) Percentage of cell death in HT‐1080 cells or human dermal fibroblasts transfected with SC or siYAP1 sequence. Cell death was assessed 3 days after cell entry into vertically confined microchannels using live–dead staining (*n* ≥ 45 cells from three independent experiments). Data are mean ± S.E.M. ** *p* ≤ 0.01. f) Representative images showing YAP1 localization in vertically confined dermal fibroblasts transfected with siLATS2. Scale bar: 5 µm. g) Nuclear to cytoplasmic ratio of YAP1 in vertically confined HT‐1080 cells or dermal fibroblasts transfected with SC or siLATS2 sequence (*n* ≥ 30 from three independent experiments). Data are mean ± SD; *p <0.05, **p < 0.01. h) Percentage of cell death in HT‐1080 cells or human dermal fibroblasts transfected with SC or siLATS2 sequence. Cell death was assessed 3 days after cell entry into vertically confined microchannels using live–dead staining (*n* ≥ 45 cells from three independent experiments). Data are mean ± S.E.M. ** *p* ≤ 0.01.

Prior work has demonstrated the key role of YAP in controlling cell life and death.^[^ ^25^ ^]^ Moreover, constitutive activation of YAP has been shown to compromise the nuclear membrane integrity.^[^ ^26^ ^]^ These findings prompted us to hypothesize that confinement‐induced YAP exit to the cytoplasm suppressed YAP‐dependent transcriptional activity, ultimately reducing the frequency of NER, P53 activity, and cell death. In agreement with this hypothesis, we demonstrated that vertically confined HT‐1080 cells that remained alive displayed reduced nuclear levels of EGFP‐YAP2 relative to those that died (**Figure** **6**a). Moreover, we found that the nuclei of vertically confined HT‐1080 cells treated with siYAP1 ruptured less frequently relative to nuclei of scramble control cells (Figure 6b). YAP1 knockdown also suppressed the fraction of fibroblasts experiencing NER events (Figure 6c) and reduced P53 activation (Figure 6d). This intervention promoted cell survival in both cell types in vertical confinement (Figure 6e and Figure S6a, Supporting Information). Conversely, transfection of HT‐1080 cells and dermal fibroblasts with an siRNA against LATS2 (Figure S6b, Supporting Information), which phosphorylated and inactivated YAP,^[^ ^27^ ^]^ markedly increased YAP1 nuclear levels (Figure 6f,g) and exacerbated confinement‐induced cell death (Figure 6h and Figure S6c, Supporting Information). Overall, our data suggest that the downregulation of YAP activity mediates cell adaptation to confinement and, thereby, cell survival.

## 3 Discussion

Herein, we develop, test, and validate a high‐throughput microfluidic platform technology that permits real‐time monitoring of single cells in physiologically relevant confined microenvironments. Our assay offers significant improvements over previous cell confinement models. First, it enables fine‐tuning of the degree and geometry of 3D confinement. This is accomplished by designing photomasks that can produce microchannels with different geometric configurations. Moreover, the PRIMO photopatterning technology allows the user to coat the microchannel surfaces with the desired ECM protein and can thus be used to create microenvironments that recapitulate the ECM composition of native tissues. The lowermost and uppermost media reservoirs are also useful in providing nutrients to confined cells and establishing a symmetric or asymmetric distribution of signals to modulate cell behavior. Furthermore, the use of a coverslip as the basal surface of the device enables high‐resolution fluorescent imaging of live or fixed cells. Our assay is innovative because it exploits the inherent capacity of migrating cells to squeeze through narrow pores, which in turn allows cell entrapment in microchannels with dimensions smaller than the size of cells.

Although confinement has been shown to reduce cell proliferation,^[^ ^13^ ^]^ little is known about its role in the regulation of cell survival. Prior studies have demonstrated that transient exposure to confinement does not compromise cell survival unless cells express low levels of the nuclear envelope protein LMNA.^[^ ^5^, ^28^ ^]^ Cell viability also remains unaffected in stiff microwells which enable prolonged cell culture in confining environments.^[^ ^8b^ ^]^ However, these microwells induce moderate confinement because they have dimensions that are larger than the average cell diameter. Here, we entrap cells in microchannels with cross‐sectional areas comparable to or smaller than the cell size. Our data reveal that constant exposure to microenvironments that physically deform the nucleus (e.g., tightly confining microchannels or 3D hydrogels with confining pores) can reduce cell viability. The environment geometry plays a key role in regulating confinement‐induced cell death. Although vertically and laterally confined microchannels have the same cross‐sectional area, only the former microchannel configuration reduces cell viability. This result, which appears to be counterintuitive, may be attributed to the top‐to‐bottom asymmetry of molecular and cytoskeletal elements (aka dorsoventral polarity).^[^ ^11a^ ^]^ In our device, cells establish dorsoventral polarity before entering confined microchannels because they adhere to a 2D‐like environment adjacent to the entrances of Collagen I‐photopatterned microchannels. We have previously shown that dorsoventral polarity exists in vivo and its elimination renders cells incapable of distinguishing between vertical and lateral confinement.^[^ ^11a^ ^]^ When dorsoventral polarity is established, vertical but not lateral confinement hyperactivates actomyosin contractility, which in turn triggers influx of cytoplasmic constituents into the nucleus, thus resulting in nuclear expansion, blebbing, and rupture.^[^ ^11^ ^]^ Of note, increased incidence of NER in vertical compared to lateral microchannels has also been observed by others.^[^ ^5a^ ^]^ Our data suggest that frequent NER events reduce cell viability in confinement, which may explain why cell death occurs primarily in vertically confined cells.

Uncontrolled exchange of material between the nucleus and the cytoplasm, as a result of NER, triggers the cytoplasmic mislocalization of diffusible DNA repair factors (e.g., Ku80 and BRCA1)^[^ ^5c^ ^]^ and the translocation into the nucleus of cytoplasmic nucleases such as TREX1.^[^ ^29^ ^]^ Although these detrimental events lead to DNA damage, mechanical stress on the nucleus can trigger DNA damage even in the absence of NER.^[^ ^15^ ^]^ This deformation‐induced DNA damage typically occurs at replication forks and appears more prominent in cell lines carrying a mutant P53 gene. Here, we demonstrate that long‐term confinement reduces cell viability in HT‐1080 cells and human fibroblasts that have a functional P53 and exhibit NER‐induced DNA damage^[^ ^15^ ^]^ but not in MDA‐MB‐231 cells that carry a mutant P53 gene and experience primarily deformation‐induced DNA damage.^[^ ^15^ ^]^ Furthermore, confined cells that die show more frequent NER compared to cells that survive. CHMP2A knockdown, which delays nuclear envelope resealing,^[^ ^5^, ^20^ ^]^ exacerbates cell death in confining microchannels, while treatment with a ROCK inhibitor, which reduces the mechanical stress on the nucleus and thus the incidence of NER,^[^ ^11b^ ^]^ abrogates confinement‐induced cell death. Thus, it is tempting to speculate that a threshold frequency value of NER exists, above which cells suffer from persistent DNA damage that leads to a sustained DNA damage response and, consequently, cell death. Consistent with this idea, we found that prolonged exposure to vertical confinement increases P53‐dependent gene expression. Importantly, cells depleted of P53 or expressing a mutant P53 gene survive in confinement. This suggests that loss‐of‐function P53 mutations, which are common in human cancers,^[^ ^30^ ^]^ may confer a survival advantage to migrating tumor cells.

Although vertical confinement reduces cell viability, not all entrapped cells die. Additionally, P53 activity displays a biphasic behavior in vertical microchannels, peaking on day 2 and returning to baseline levels by day 3. These results suggest that migrating cells can modify their intracellular mechanisms to adapt to confinement. The mechanotransducer YAP plays a key role in this mechanoadaptation process. We find that YAP1 and YAP2 escape the nucleus in vertical but not in partial confinement, and this translocation event triggers a reduction in YAP transcriptional activity. Confinement‐induced NER events fail to alter the subcellular distribution of YAP. Instead, YAP nuclear exit is regulated at least in part by Exportin 1. Although the underlying mechanism remains unclear, recent findings have demonstrated that nucleocytoplasmic transport is mechanosensitive.^[^ ^31^ ^]^ Thus, it is likely that confinement may change the conformation of nuclear pore complexes in such a way that facilitates the exit of nuclear proteins to the cytoplasm.^[^ ^32^ ^]^ Confinement may also disrupt the RAN gradient, which controls the directionality of nucleocytoplasmic transport.^[^ ^33^ ^]^ Such a disruption may promote the nuclear export of YAP or interfere with its import. Regardless of the underlying mechanisms, YAP exit to the cytoplasm is associated with a high likelihood of survival, while LATS2 knockdown, which triggers YAP accumulation in the nucleus, exacerbates cell death in confinement. Our study suggests that downregulation of YAP activity supports cell survival by suppressing the frequency of NER events and restricting P53 activation. This is consistent with reports showing that YAP activation increases the expression of P53^[^ ^34^ ^]^ and actomyosin contractility (e.g., by upregulating MYL9 or RhoGEFs),^[^ ^35^ ^]^ which drives nuclear deformation, blebbing, and rupture.^[^ ^11^, ^36^ ^]^

In summary, using sophisticated microfluidic assays and imaging tools, we demonstrate that the geometry and the degree of confinement control the growth and survival of migrating cells. We also show that the tumor suppressor P53 is a key factor governing cell life and death in confining microenvironments. Last, we report the critical role of YAP in regulating NER, and P53 activity and thereby mediating cell adaptation to confinement. Our work has broader implications in cancer metastasis since it generates new knowledge on how migrating tumor cells sense and respond to long‐term 3D confinement. It has been shown that entrapment of circulating tumor cells in confining microvessels is associated with reduced cell viability.^[^ ^1^, ^6^ ^]^ Thus, developing strategies to enhance cell responsiveness to confinement may represent a novel approach to inhibit intravascular tumor cell survival.

## 4 Experimental Section

### Cell Culture and Treatment with Chemical Inhibitors or siRNAs

Human dermal fibroblasts (GM05565) and MDA‐MB‐231 breast cancer cells (NCI‐PBCF‐HTB26) were purchased from the Coriell Institute and the American Type Culture Collection, respectively. HT‐1080 fibrosarcoma cells, NLS‐mCherry‐tagged HT‐1080 cells, and LifeAct‐GFP and H2B‐mCherry‐labeled HT‐1080 cells were a gift from K. Konstantopoulos (Johns Hopkins University). All cell lines were cultured in Dulbecco's modified Eagle's medium (DMEM; Gibco) supplemented with 10% v/v FBS (Bio‐Techne) and 1% v/v penicillin/streptomycin (Gibco), maintained at 37 °C and 5% CO_2_ and passaged every 2 to 5 days. Experiments with inhibitors were performed using cells treated with Y‐27632 (MilliporeSigma, 10 µm), pifithrin‐alfa (Enzo; 20 µm), puromycin (VWR; 1 µg mL^−1^), selinexor (KPT‐330; 10 µm), GM 6001 (MilliporeSigma; 10 µm), or corresponding vehicle controls. For gene knockdown experiments, cells were transfected with YAP1 siRNA (sc‐38637; Santa Cruz Biotechnology), P53 siRNA (sc‐29435; Santa Cruz Biotechnology), LATS2 siRNA (sc‐37444; Santa Cruz Biotechnology), CHMP2A siRNA (sc‐60369; Santa Cruz Biotechnology), or scramble siRNA (sc‐37007; Santa Cruz Biotechnology) using the Lipofectamine RNAiMax Kit (Thermo Fisher Scientific). The transfection of NLS‐mCherry‐tagged HT‐1080 cells with EGFP‐YAP2 was carried out using Lipofectamine 3000 transfection Kit (Thermo Fisher Scientific).

### Cloning, Lentivirus Preparation, and Transduction

The EGFP‐YAP2 plasmid and the P53‐RE LVDP constructs were generously given by Dr. Jho and Dr. Andreadis, respectively. YAP/TEAD‐RE LVDP vector was generated by subcloning ten repeats of YAP/TEAD‐RE (ACATTCCA) into LVDP using BsiWI and BstBI as restriction sites. The cloned product was verified by Sanger Sequencing. To produce lentiviral particles, HEK 293T/17 cells were cotransfected with psPAX2 (Addgene; plasmid #12 260), pMD2.G (Addgene, plasmid #12 259), and P53‐RE or YAP/TEAD‐RE LVDP vectors using the calcium phosphate precipitation method. 48 h later, cell culture supernatant containing lentiviral particles was harvested, filtered through 0.45 µm filters and centrifuged at 50 000 × *g* for 2 h at 4 °C. The virus pellet was then resuspended in cell culture medium. Cell transduction was carried out by mixing the concentrated virus with growth medium containing 8 µg mL^−1^ polybrene (AmericanBio).

### Fabrication and Photopatterning of Microfluidic Devices

PDMS‐based microfluidic devices, which consisted of two large, 2D‐like channels and an array of parallel microchannels of constant length (*L*= 200 µm) but varying width or height (10 or 3 µm), were fabricated using standard multilayer photolithography and replica molding as described previously.^[^ ^11^ ^]^ The exact dimensions of partially, vertically, and laterally confined microchannels were *W* × *H* = 11.3 × 10.7 µm^2^, *W* × *H* = 9.8 × 3.1 µm^2^, and *W* × *H* = 2.9 × 9.9 µm^2^, respectively. To deposit ECM molecules exclusively on the microchannel walls, the PRIMO photopatterning technology^[^ ^12^ ^]^ (Alveole) was utilized. The devices were first treated with 0.1% v/v PLL (MilliporeSigma) for 1 h followed by washing with 10 mm HEPES buffer (pH = 8–8.5). Next, the devices were coated overnight with 50 mg mL^−1^ mPEG‐SVA (5 kD; Laysan Bio) to create an antifouling layer that prevented ECM deposition. Microscopy, UV light (375 nm), and the photoactivatable reagent PLPP (Alveole) were then employed to cleave the anti‐adhesive layer that lined the microchannel walls. Subsequently, microchannels were coated with Collagen I‐FITC (5–20 µg mL^−1^; MilliporeSigma) or rat tail Collagen I (20 µg mL^−1^; Gibco) solutions for 1 h at 37 °C. Devices or microchannels treated only with PLL‐mPEG‐SVA served as a negative control. Collagen I‐coated devices were prepared as described previously.^[^ ^11^ ^]^ Collagen I‐FITC deposition was examined using a Nikon Ti2 Inverted Microscope or a Nikon A1 confocal microscope.

### Cell Seeding in Microfluidic Devices and Live‐Cell Imaging

20 µL of cell suspension (3 × 10^6^ cells per mL) was added to the inlet well (Figure 1e), generating cell flow through the lowermost 2D‐like channel. Devices were then incubated for 15 min at 37 °C and 5% CO_2_ to allow cells to adhere adjacent to the microchannel entrances. Next, the bottom wells of the devices were filled with DMEM supplemented with 1% v/v penicillin/streptomycin and the top wells with DMEM supplemented with 10% FBS v/v and 1% v/v penicillin/streptomycin to create a chemotactic gradient. After cell entry into microchannels, the chemotactic gradient was removed and cells were cultured in DMEM supplemented with 10% FBS v/v and 1% v/v penicillin/streptomycin. The culture medium was replenished every ≈24 h to avoid issues arising from evaporation or nutrient depletion. Microchannels containing more than one cell were excluded from subsequent analysis.

Cell imaging was carried out using a Nikon Ti2 Inverted Microscope equipped with a Tokai Stage‐Top Incubator that enables control of CO_2_, temperature, and humidity. During imaging, cells were maintained at 37 °C and 5% CO_2_. Fluorescent images were taken using GFP, TRITC, or DAPI filters.

### Quantification of Cell Entry, Cell Entrapment, Cell Division, Cell Migration, and Cell Death

The percentage of cell entry was calculated by dividing the number of cells that completely entered the microchannels 2 h or 18 h after cell seeding by the total number of cells seeded adjacent to microchannel entrances. Cell entrapment was assessed by quantifying the fraction of cells that remained inside microchannels following cell entry. The MTrackJ plugin was employed to track cell movement in microchannels as previously described.^[^ ^11a^ ^]^ Migration velocity was calculated by measuring cell displacement divided by the total time cells spent in microchannels. Dividing and non‐viable cells were excluded from the calculation of cell entrapment and migration velocity. Cell division was quantified on days 1, 2, and 3 by calculating the fraction of cells that divided inside different microchannel configurations. Cell entry, cell entrapment, and cell division were monitored using time‐lapse microscopy. Cell viability in microchannels was evaluated on day 3 using live–dead staining (Biovision) according to the manufacturer's instructions. Specifically, cell viability was quantified by counting the total number of live cells (green) and dead cells (red) in each image. Cell and nuclear blebbing were tabulated manually using fibroblasts stained with Alexa Fluor 488 Phalloidin (cytoskeleton) and Hoechst (nucleus) or HT‐1080 cells labeled with LifeAct‐GFP and NLS‐mCherry. Quantification was carried out at a fixed time point (24 or 72 h).

### Quantification of P53‐RE and YAP/TEAD‐RE

Time‐lapse fluorescent imaging was performed for 2 or 3 days (≈24 h intervals) using GFP and TRITC filters. Image analysis was conducted using the ImageJ software (National Institute of Health). The GFI and RFI were measured for each cell inside the microchannels at all‐time points using the following formula

(1) $$\frac{\mathrm{GFI}}{\mathrm{RFI}}=\frac{\mathrm{Mean}\;\mathrm{GFI}\;\mathrm{of}\;\mathrm{cell}-\mathrm{mean}\;\mathrm{GFI}\;\mathrm{of}\;\mathrm{background}}{\mathrm{Mean}\;\mathrm{RFI}\;\mathrm{of}\;\mathrm{cell}-\mathrm{mean}\;\mathrm{RFI}\;\mathrm{of}\;\mathrm{background}}\;\mathrm{The}\;\mathrm{ratio}\;\mathrm{GFI}/\mathrm{RFI}\;\mathrm{represented}\;\mathrm{the}\;\mathrm{transcriptional}\;\mathrm{activity}\;\mathrm{of}\;P\;53\;\mathrm{or}\;\mathrm{YAP}/\mathrm{TEAD}$$

### Caspase‐3/7 Activity Measurements

The NucView 488 caspase‐3 substrate (Biotium) solution was prepared by dissolving the substrate in culture medium at a concentration of 1 µm. Cells already confined for 48 h were treated with this solution, and fluorescent images were taken every 20 min for 12 h. Image quantification was carried out using ImageJ. The normalized Caspase‐3/7 activity was calculated using the following formula

(2) $$\mathrm{Normalized}\;\mathrm{Caspase}3/7\;\mathrm{activity}=\frac{\mathrm{Mean}\;\mathrm{GFI}\;\mathrm{of}\;\mathrm{cell}\;\left(t=12\;h\;\mathrm{or}\;\mathrm{before}\;\mathrm{cell}\;\mathrm{death}\right)-\mathrm{Mean}\;\mathrm{GFI}\;\mathrm{of}\;\mathrm{background}}{\mathrm{Mean}\;\mathrm{GFI}\;\mathrm{of}\;\mathrm{cell}\;\left(t=0\;h\right)-\mathrm{Mean}\;\mathrm{GFI}\;\mathrm{of}\;\mathrm{background}}\;$$

### BSA‐FITC Diffusion through Vertically Confined Microchannels

To determine if vertically confined cells have access to nutrients, growth medium containing BSA‐FITC (2 mg mL^−1^; Rockland) was added to the device, and fluorescent images were taken every 5 min over a period of 3 h using a GFP filter.

### Immunocytochemistry

Cells were fixed with 4% paraformaldehyde (Affymetrix Inc.) for 10 min, followed by permeabilization with 0.1% Triton X‐100 (MilliporeSigma) for 15 min and blocking for 1 h with a solution that contained 1% BSA (MilliporeSigma), 2% goat serum (Vector Laboratories), and 0.1% Triton X‐100. Next, anti‐Yap1 (1:50, Santa Cruz Biotechnology) and anti‐Histone H3 (1:400, Cell Signaling) antibodies were applied to cells overnight at 4 °C. Cells were then incubated with Alexa Flour 488 goat anti‐rabbit (1:100, Thermo Fisher Scientific), Alexa Flour 568 goat‐anti mouse (1:200, Thermo Fisher Scientific), and Hoechst 33 342 (1:2500, Biotium) for 2 h. Primary and secondary antibodies were dissolved in 1% BSA (MilliporeSigma)/0.3% Triton X‐100 (MilliporeSigma). Samples incubated only with secondary antibodies served as negative controls. For Phalloidin staining, cells were blocked with a solution containing 5% goat serum and 0.1% Triton X‐100, and stained with Phalloidin (1:100, Thermo Fisher Scientific) and Hoechst 33 342 (1:2500, Biotium) for 1 h.

### YAP1, Histone H3, and EGFP‐YAP2 Quantification

To calculate YAP1, Histone H3, or EGFP‐YAP2 nuclear to cytoplasm ratio, the following formula was used

(3) $$\frac{\mathrm{Nuclear}}{\mathrm{Cytoplasmic}}=\frac{\mathrm{Mean}\;\mathrm{fluorescence}\;\mathrm{intensity}\;\mathrm{of}\;\mathrm{the}\;\mathrm{nucleus}-\mathrm{Mean}\;\mathrm{fluorescence}\;\mathrm{intensity}\;\mathrm{of}\;\mathrm{background}}{\mathrm{Mean}\;\mathrm{fluorescence}\;\mathrm{intensity}\;\mathrm{of}\;\mathrm{the}\;\mathrm{cytoplasm}-\mathrm{Mean}\;\mathrm{fluorescence}\;\mathrm{intensity}\;\mathrm{of}\;\mathrm{background}}$$

### Western Blot

Western blot was performed to measure CHMP2A and LATS2 protein content in HT‐1080 cells or dermal fibroblasts under control or knockdown conditions. Briefly, total protein was extracted from the cell lysates using RIPA buffer (Thermo Fisher Scientific) containing a protease inhibitor cocktail (Roche). Protein concentrations were measured using the BSA protein assay kit (Thermo Fisher Scientific) and equal amounts of protein (20 and 50 µg, for CHMP2A and LATS2, respectively) were separated by SDS‐PAGE using a 4–15% polyacrylamide gel. The proteins were then transferred onto a PVDF membrane (Bio‐Rad Laboratories) using the Trans‐Blot Turbo Transfer System (Bio‐Rad Laboratories) or the wet transfer method. The membrane was blocked with 5% non‐fat milk in tris‐buffered saline with 0.1% Tween‐20 (TBST) for 1 h at room temperature and then incubated with primary antibodies against protein LATS2 (Abcam, 1:1000) and CHMP2A (Proteintech; 1:1000) overnight at 4 °C. After washing with TBST, the membrane was incubated with a secondary antibody conjugated to horseradish peroxidase (Cell signaling; 1:2000 dilution) for 1 h at room temperature. Protein bands were visualized using the ECL substrate (Thermo Fisher Scientific).

### Collagen‐Based Hydrogel Preparation

Methacrylated collagen (collagen‐MA) was purchased from Advanced Biomatrix. Methacrylated hyaluronic acid (HA‐MA) was synthesized following previously reported methods^[^ ^37^ ^]^ and dissolved in 10 mm HEPES buffer containing 150 mm NaCl at 20 mg mL^−1^. Irgacure 2959 (MilliporeSigma) was dissolved in methanol at 100 mg mL^−1^. Soft and stiff hydrogels were prepared by mixing collagen‐MA (2.5 to 6 mg mL^−1^) and methacrylated hyaluronic acid (0 to 1 mg mL^−1^) at various densities. To provide chemically crosslinked networks through methacrylate groups, 1.9 mg mL^−1^ of Irgacure 2959 (MilliporeSigma) was added only to the stiff hydrogel mixtures. For the soft hydrogels, the same volume of HEPES buffer and methanol was added to the hydrogel mixture as in the stiff hydrogel condition. The mixture of collagen‐MA, HA‐MA, and Irgacure solution was neutralized to pH 7.4 using neutralization solution (Advanced Biomatrix), and hydrogels were allowed to polymerize at 37 °C for 30 min. After gelation, all hydrogels were exposed to UV light for 300 s.

### Cell Encapsulation and 3D Culture

All hydrogel precursors were mixed homogeneously before being combined with cell suspension. HT‐1080 was encapsulated at 0.5 × 10^6^ cells per mL. After UV exposure, the cell‐laden constructs were washed with DMEM media containing 0.2% dimethyl sulfoxide (DMSO) for 1 h, followed by GM 6001 treatment. The cell culture media was supplemented with 10 µm of GM 6001 or the vehicle control (DMSO) and exchanged every 24 h up to day 3.

### Cell Viability in Hydrogels

Cell viability in hydrogels was analyzed by live–dead staining using calcein‐AM (1:1000 dilution), ethidium homodimer (1:500 dilution), and Hoechst 33 342 (1:200 dilution) on days 1 and 3. Confocal images were captured as 50 µm z‐stacks with 3.171 µm slices using a Nikon eclipse Ti2 with a 10 × objective lens. Cell viability was quantified using ImageJ by counting the total number of live cells (stained by calcein‐AM) and dead cells (stained by ethidium homodimer) in each image of maximum intensity projection produced by NIS‐elements AR software.

### Oscillatory Rheology

Hydrogel viscoelasticity was analyzed using a Discovery Hybrid Rheometer (HR20; TA Instruments) equipped with an 8 mm cross‐hatched geometry and 500 µm gap size at 37 °C.^[^ ^37^ ^]^ A premade hydrogel disk (140 µL, 8 mm in diameter) was placed on a plate/cross‐hatched geometry, and PBS was added around the geometry to ensure the hydrogels remained swollen. An amplitude sweep was performed from 0.1% to 10% at 0.25 Hz. Values of storage and loss moduli were reported from measurements within the linear viscoelastic regime at 0.5%. Young's modulus (*E*) was calculated from the shear modulus through the relationship: *E* = 2*G*(1+*ν*) (*E* is Young's modulus, *G* is shear modulus and *ν* is Poisson's ratio), assuming a Poisson ratio of 0.5.^[^ ^38^ ^]^

### Matrix Pore Size Measurement

Hydrogel pore cross‐section measurement was performed as described previously.^[^ ^5^, ^39^ ^]^ Briefly, soft and stiff hydrogel constructs were prepared and the pore dimensions were obtained from 30 µm z stacks with 0.2 µm slices of acellular hydrogel substrates. The reflective image was orthogonally reconstructed to *XZ* plane images. The pore size was quantified by Fiji ImageJ software using the area measurements with the same threshold setting across all images. Watershed segmentation was performed to produce closed pores in XZ plane images.

### Preparation of Polyacrylamide Gels and Stiffness Measurements

Coverslips were exposed to heat (1080 °F), treated with NaOH (0.1 n), and activated with glutaraldehyde (0.5% v/v). Next, a prepolymer solution was prepared to contain acrylamide (A) (40%; Bio‐Rad Laboratories), *N*,*N*′‐methylene‐bis‐acrylamide (B) (2%; Bio‐Rad Laboratories), 10% ammonium persulfate (Bio‐Rad Laboratories), *N*,*N*,*N*′,*N*′‐tetramethylethylenediamine (Bio‐Rad Laboratories), and HEPES (VWR). For 0.2 kPa gels, 3% A/0.2% B was used while 8% A/0.6% B was used for 12 kPa PAM gel. The prepolymer solution was deposited on the activated coverslips and allowed to polymerize for 30 min. To coat the polyacrylamide surface with ECM molecules, hydrogels were treated with sulfosuccinimidyl‐6‐(4′‐azido‐2′‐nitrophenylamino)hexanoate (sulfo‐SANPAH) (1 mg mL^−1^), exposed to UV light (wavelength 254 nm) for 15 min, and incubated with rat tail Collagen I or Collagen I‐FITC (20 µg mL^−1^) at 37 °C for 1 h. The fluorescence intensity of deposited Collagen I‐FITC was measured using ImageJ.

### Stiffness Measurements of Polyacrylamide Gels

Rings of polyacrylamide gels with radial wall thickness of ≈2 mm and height of ≈2 mm were mounted on a force transducer (ADInstruments) and pulled gradually until failure. The recorded force was normalized to the cross‐sectional area of each ring to derive stress values. The dimensions of rings were measured using a digital vernier caliper and strain was calculated by dividing the change in length by the initial length of the ring. The Young's Modulus was derived from the slope of the linear portion of the stress–strain curve.

### Statistical Analysis

Unless otherwise stated, experiments were performed at least three independent times. The following statistical tests were used to determine statistical significance (*p* < 0.05): a) Student's *t*‐test for comparing two groups, b) one‐way analysis of variance (ANOVA) test followed by a Tukey's test for multiple comparisons, and c) two‐way ANOVA for experiments with two variables and more than two groups. Analysis was performed using GraphPad Prism 6, 7, or 8 software.

## Conflict of Interest

The authors declare no conflict of interest.

## Author Contributions

F.H. designed the study, performed most experiments, analyzed data, and wrote the manuscript. A.A., F.A., A.T., A.M., and C.M. performed select experiments and analyzed data. A.Af., helped with microfabrication. A.V.A. helped in Western blotting. S.A. provided the LVDP vector. V.B. helped in Western blotting, performed select confocal microscopy experiments, provided critical input, and edited the manuscript. J.S. and S.G. performed experiments in collagen‐based hydrogels, provided critical input, and edited the manuscript. P.M. designed and supervised the study and wrote the manuscript.

## Supporting information

Supporting Information

Supplemental Video 1

Supplemental Video 2

Supplemental Video 3

## Data Availability

The data that support the findings of this study are available from the corresponding author upon reasonable request.
