# Supplementary material for: Downregulation of YAP Activity Restricts P53 Hyperactivation to Promote Cell Survival in Confinement
Source: Adv Sci (Weinh). 2023 Jun 2;10(23):2302228. doi: 10.1002/advs.202302228 (PMC10427377; doi:10.1002/advs.202302228)
Supplement: Supplementary file 1 — Supporting Information [file ADVS-10-2302228-s004.pdf]

ADVANCED SCIENCE
Open Access

Supporting Information

for *Adv. Sci.*, DOI 10.1002/advs.202302228

Downregulation of YAP Activity Restricts P53 Hyperactivation to Promote Cell Survival in Confinement

*Farnaz Hemmati, Ayuba Akinpelu, Jiyeon Song, Farshad Amiri, Anya McDaniel, Collins McMurray, Alexandros Afthinos, Stelios T. Andreadis, Andrew V. Aitken, Vinicia C. Biancardi, Sharon Gerecht and Panagiotis Mistriotis**

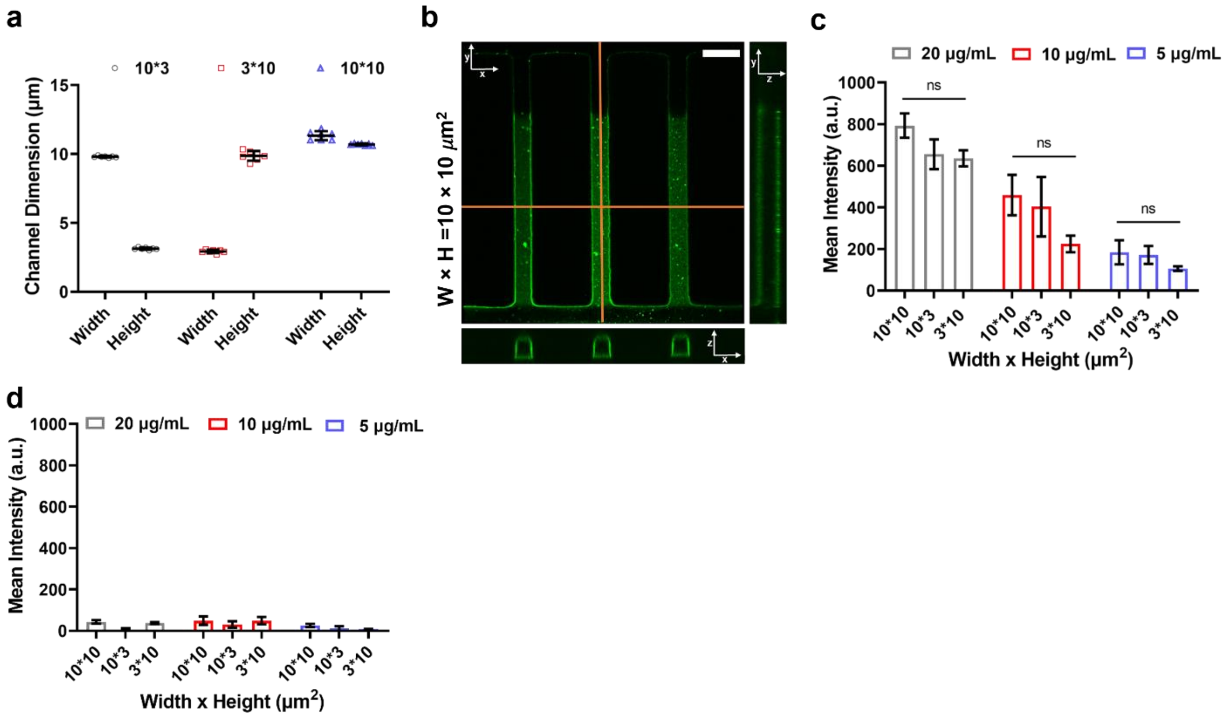

Supplementary Figure 1. (a) Dimensions of partially, vertically and laterally confined microchannels, as measured by a profilometer (n = 10 channels). Data are mean ± S.D. **(b)** Representative confocal images (XY, YZ and XZ projections) of three partially confined microchannels coated with Collagen I-FITC (20 µg/mL). Scale bar: 20 µm. **(c)** Same data as in **Figure 1J** replotted to show that at a given Collagen I-FITC concentration, the deposited Collagen I-FITC was similar between different microchannel geometries (n≥ 40 channels from at least 4 independent experiments). Data are mean ± S.E.M **(d)** Mean intensity of Collagen I-FITC photopatterned on the walls of different PEGylated microchannels. Collagen I-FITC concentration: 5-20 µg/mL. Data are mean ± S.E.M.

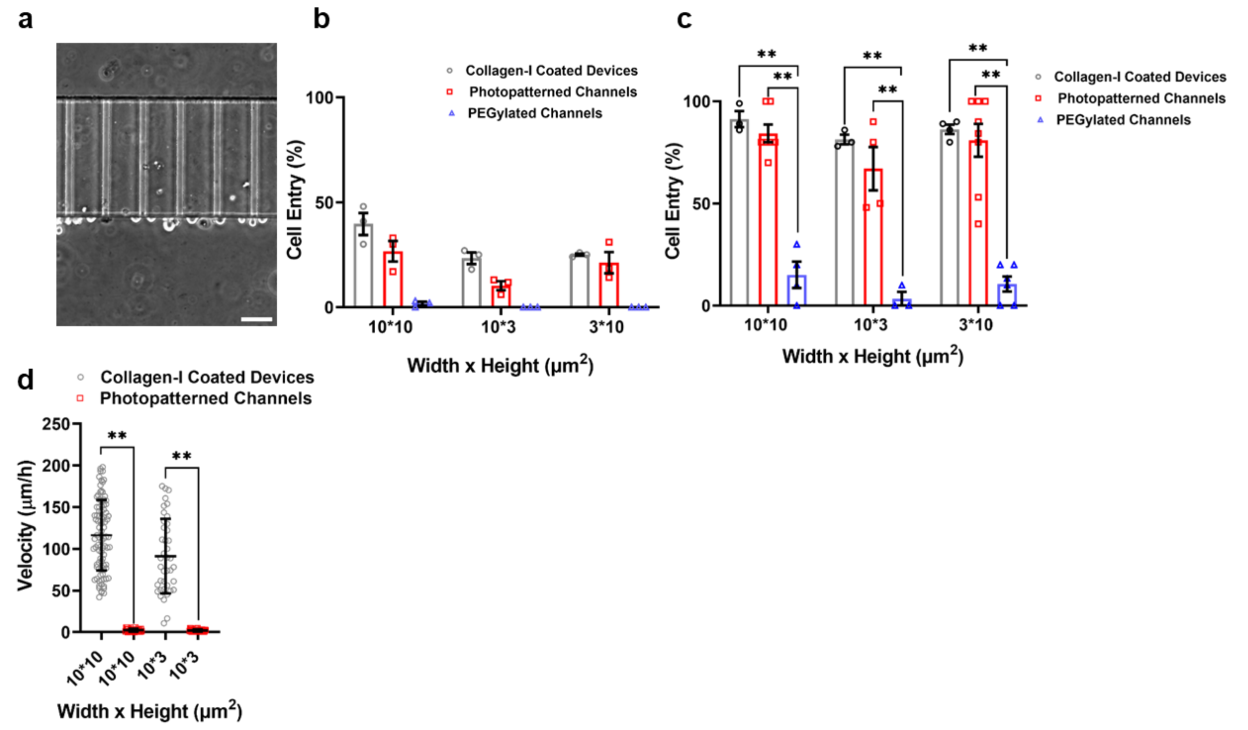

Supplementary Figure 2. (a) A representative image showing HT-1080 cells adhering adjacent to the entrances of vertically confined microchannels photopatterned with Collagen I (20 μg/mL). Scale bar: 50 μm**. (b)** Percentage of cell entry into partially, vertically and laterally confined microchannels coated with Collagen I (20 μg/mL) two hours after cell seeding and in the absence of chemoattractant molecules. Collagen I-coated devices and PEGylated devices served as positive and negative controls, respectively (n≥ 45 cells from 3 independent experiments). Data are mean ± S.E.M. **(c)** Percentage of cell entry into partially, vertically and laterally confined microchannels coated with Collagen I (20 μg/mL) 18 hours after cell seeding and in the presence of a chemotactic gradient. Collagen I-coated devices and PEGylated devices served as positive and negative controls, respectively (n≥ 45 cells from 3 independent experiments). Data are mean ± S.E.M. **p≤ 0.01. **(d)** Velocity of human HT-1080 in partially or vertically confined microchannels photopatterned with 20 μg/mL Collagen I. Collagen I-coated devices served as a control (n≥ 45 cells from 3 independent experiments). Data are mean ± S.D. **p≤ 0.01.

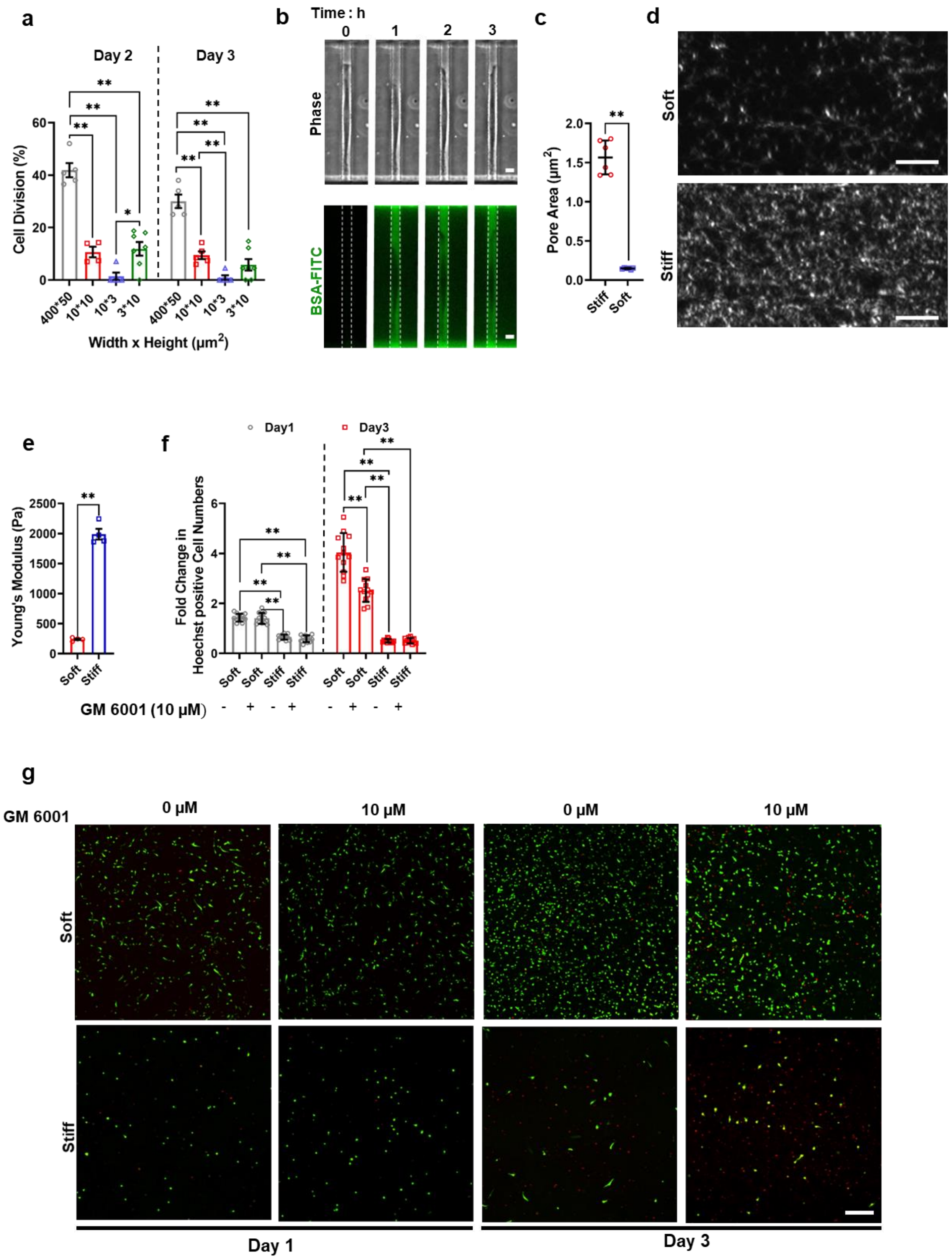
a
Day 2
Day 3
Cell Division (%)
Width x Height (μm2)
400*50
10*10
10*3
3*10
b
Time : h
0
1
2
3
Phase
BSA-FITC
c
Pore Area (μm2)
Stiff
Soft
d
Soft
Stiff
e
Young's Modulus (Pa)
Soft
Stiff
f
Day1
Day3
Fold Change in Hoechst positive Cell Numbers
Soft
Stiff
GM 6001 (10 μM)
g
GM 6001
0 μM
10 μM
Soft
Stiff
Day 1
Day 3

Supplementary Figure 3. (a) Percentage of HT-1080 cells that divided on 2D (400 x 50 μm^2 channels) or inside partially, vertically and laterally confined microchannels on days two and three (n≥ 45 cells from at least 4 independent experiments). Data are mean ± S.E.M**.** **p≤ 0.01 **(b)** Image sequence showing diffusion of BSA-FITC around a vertically confined cell. Scale bar: 10 µm. **(c)** Pore size measurements of soft or stiff methacrylated collagen hydrogels (n=6 independent samples). Data are mean ± S.E.M. **p≤ 0.01. **(d)** Representative images showing the porosity of soft and stiff hydrogels. Scale bar: 5 µm. **(e)** Stiffness measurements of soft or stiff methacrylated collagen hydrogels (n=4 independent samples). Data are mean ± S.E.M**.** **p≤ 0.01. **(f)** Fold increase in cell numbers measured by the number of Hoechst positive HT-1080 cells in soft or stiff methacrylated collagen hydrogels on days one and three. Cells were treated with an MMP inhibitor (GM 6001; 10 µM) or vehicle control (n≥500 cells from 3 independent experiments). Data are mean ± S.D. **p≤ 0.01. **(g)** Representative images of cells encapsulated in soft or stiff methacrylated collagen hydrogels on days one or three. Cells were treated with an MMP inhibitor (GM 6001; 10 µM) or vehicle control and stained with Live/Dead stain. Scale bar: 250 µm.

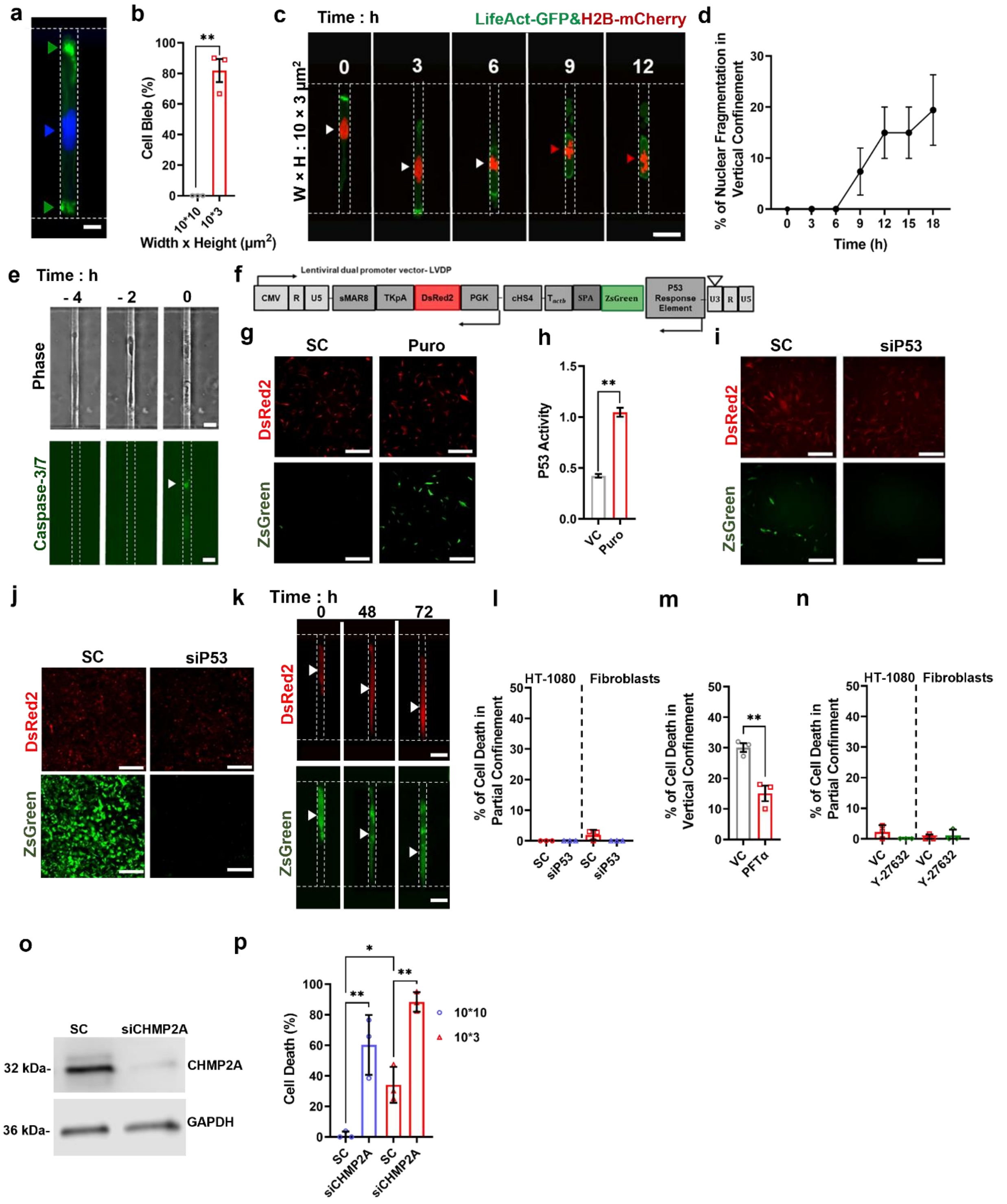

Supplementary Figure 4. (a) A representative image of a blebbing fibroblast fixed and stained with Phalloidin (green) and Hoechst (blue) in vertically confined microchannel. The green arrowhead shows membrane blebs and the blue arrowhead shows a nucleus experiencing

nuclear blebbing. Scale bar: 10 μm. **(b)** Percentage of LifeAct-GFP and H2B-mCherry-labeled HT-1080 cell exhibiting cell blebbing in partially versus vertically confined microchannels (n≥ 30 cells from 3 independent experiments). Data are mean ± S.E.M. **p≤ 0.01. **(c)** Image sequence of LifeAct-GFP- and H2B-mCherry–labeled HT-1080 cell experiencing nuclear fragmentation in vertical microchannel. The white and red arrowheads show non-fragmented and fragmented nuclei, respectively. Scale bar: 50 μm. **(d)** Percentage of vertically confined LifeAct-GFP- and H2B-mCherry–labeled HT-1080 fibrosarcoma cells experiencing nuclear fragmentation as a function of time (n≥ 15 cells from 3 independent experiments). Data are mean ± S.E.M **(e)** Image sequence of a dermal fibroblast activating Caspase-3/7 in a vertical microchannel. Caspase-3/7 activity was visualized using the NucView dye. The white arrowhead shows Caspase-3/7 activation. Scale bar: 10 μm. **(f)** Schematic representation of the lentiviral dual promoter vector used to measure P53 transcriptional activity (modified from[17a]). **(g)** Representative images showing p53 activity in dermal fibroblasts treated with VC or puromycin (1 μg/mL). Scale bar: 100 μm. **(h)** Quantification of **(g)** (n≥ 30 cells from 3 independent experiments). Data are mean ± S.E.M. **p< 0.01. **(i, j)** Representative images showing P53 activity in **(i)** dermal fibroblasts and **(j)** HT-1080 cells transfected with a SC or siP53 sequence. Scale bar: 100 μm. **(k)** Image sequence showing a vertically confined dermal fibroblast that did not activate P53. Scale bar: 20 μm. **(l)** Percentage of cell death in partially confined HT-1080 cells or dermal fibroblasts transfected with SC or siP53 sequence. Cell death was assessed 3 days after cell entry into microchannels using live-dead staining (n≥ 45 cells from 3 independent experiments). **(m)** Percentage of cell death in vertically confined dermal fibroblasts treated with VC or P53 inhibitor (20 μM; Pifithrin-alfa (PFTα)). Cell death was assessed 3 days after cell entry into microchannels using live-dead staining (n≥ 45 cells from 3 independent experiments). Data are mean ± S.E.M. **p≤ 0.01. **(n)** Percentage of cell death in partially confined HT-1080 cells or dermal fibroblasts treated with VC or Y-27632 (10 μM). Cell death was assessed 3 days after cell entry into microchannels using live-dead staining (n≥ 45 cells from 3 independent experiments). **(o)** Western

blot showing the knockdown efficiency of siCHMP2A in dermal fibroblasts (n = 2 independent experiments). **(p)** Percentage of cell death in partially and vertically confined dermal fibroblasts transfected with a SC or siCHMP2A sequence. Cell death was assessed 3 days after cell entry into microchannels using live-dead staining ($n \geq 45$ cells from 3 independent experiments). *$p<0.05$, **$p< 0.01$.

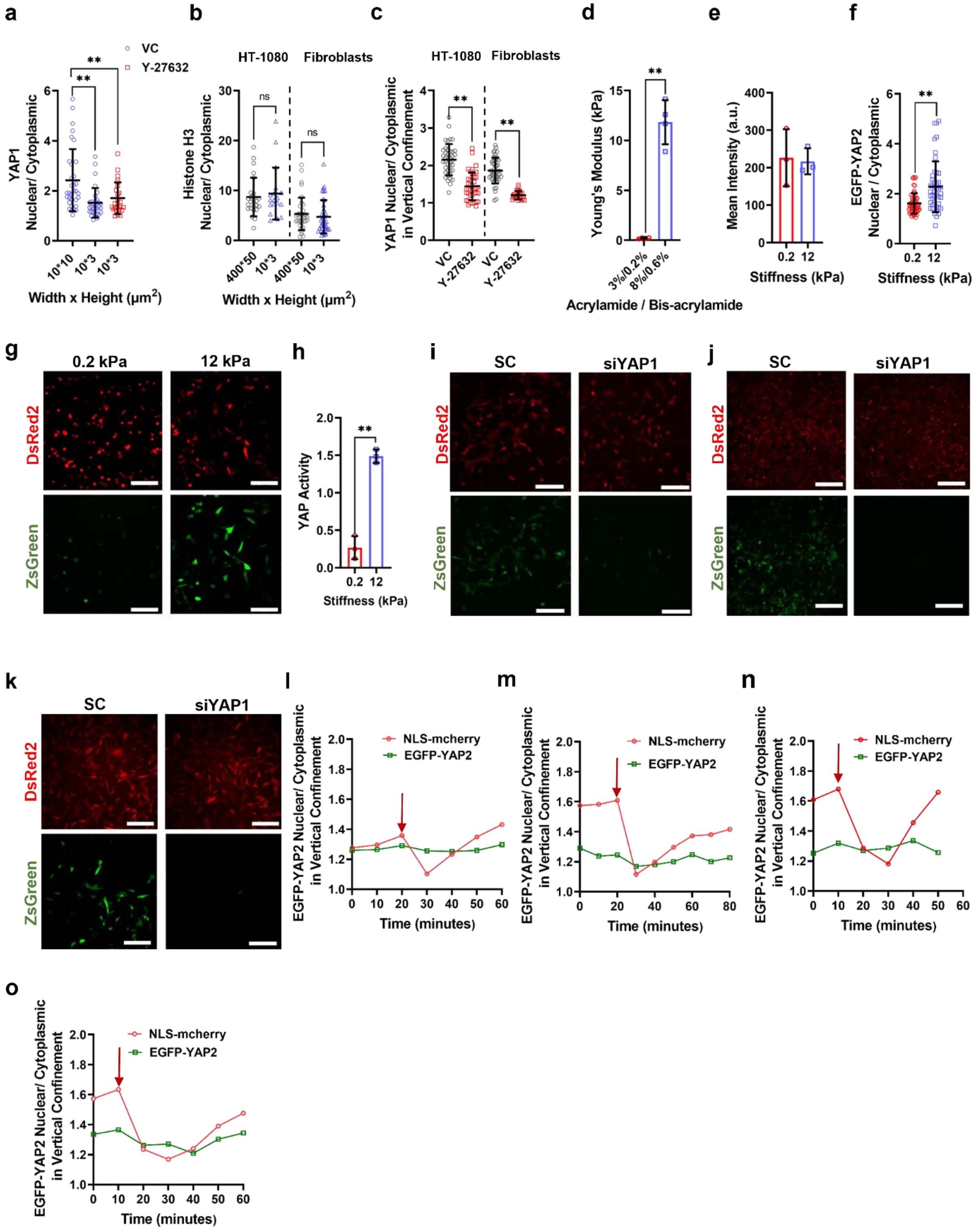

Supplementary Figure 5. (a) Nuclear to cytoplasmic ratio of YAP1 in dermal fibroblasts after 24 hours of entrapment into partially and vertically confined microchannels. Cells were treated with

VC or ROCK inhibitor (10 μM; Y-27632). (n≥45 cells from 3 independent experiments). Data are mean ± S.D. **p< 0.01. **(b)** Nuclear to cytoplasmic ratio of Histone H3 in dermal fibroblasts and HT-1080 cells entrapped in vertically confined microchannels or cultured on 2D surfaces (400 x 50 μm^2 channels) (n≥ 30 cells from 3 independent experiments). **(c)** Nuclear to cytoplasmic ratio of YAP1 in HT-1080 cells and dermal fibroblasts cultured on 2D surfaces (400 x 50 μm^2 channels) and treated with VC or Y-27632 (10 μM) (n≥ 45 cells from 3 independent experiments). **p< 0.01. **(d)** Stiffness measurements of polyacrylamide gels prepared using different Acrylamide to Bis-acrylamide ratios (n≥ 14 samples from at least 3 independent experiments). **(e)** Mean intensity of Collagen-type I FITC deposited on 0.2 and 12 kPa hydrogels (3 independent experiments). **(f)** Nuclear to cytoplasmic ratio of EGFP-YAP2 in HT-1080 cells cultured on 0.2 and 12 kPa polyacrylamide hydrogels (n≥45 cells from 3 independent experiments). **p< 0.01. **(g)** Representative images showing YAP-RE activity in MDA-MB-231 breast cancer cells cultured on soft (0.2 kPa) or stiff (12 kPa) hydrogels. Scale bar: 100 μm. **(h)** Quantification of **(g)** (n≥ 30 cells from 3 independent experiments). Data are mean ± S.E.M. **p< 0.01. **(i, j, k)** Representative images showing YAP activity in **(i)** MDA-MB-231 cells, **(j)** HT-1080 cells and **(k)** dermal fibroblasts transfected with a SC or siYAP1 sequence. Scale bar: 100 μm. **(l-o)** Four additional examples showing the nuclear to cytoplasmic ratio of NLS-mCherry and EGFP-YAP2 in vertically confined, KPT-330-treated HT-1080 cells as they experience NER events.

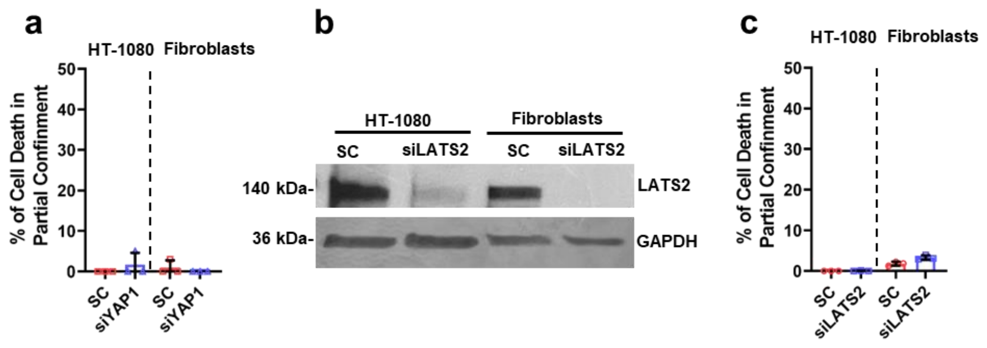

Supplementary Figure 6. (a) Percentage of cell death in partially confined HT-1080 cells or dermal fibroblasts transfected with a SC or siYAP1 sequence. Cell death was assessed 3 days after cell entry into microchannels using live-dead staining (n≥ 45 cells from 3 independent experiments). **(b)** Western blot showing the knockdown efficiency of siLATS2 in HT-1080 cells and dermal fibroblasts (n = 2 independent experiments). **(c)** Percentage of cell death in partially confined HT-1080 cells or dermal fibroblasts transfected with a SC or siLATS2 sequence. Cell death was assessed 3 days after cell entry into microchannels using live-dead staining (n≥ 45 cells from 3 independent experiments).
